# Taurine Ameliorates Tunicamycin-Induced Liver Injury by Disrupting the Vicious Cycle between Oxidative Stress and Endoplasmic Reticulum Stress

**DOI:** 10.3390/life12030354

**Published:** 2022-02-28

**Authors:** Sou Hyun Kim, Hyeji Seo, Doyoung Kwon, Dong Yeon Yuk, Young-Suk Jung

**Affiliations:** 1 Department of Pharmacy, College of Pharmacy, Pusan National University, Busan 46241, Korea; souhyun@pusan.ac.kr (S.H.K.); hseo9393@pusan.ac.kr (H.S.); 2 Research Institute for Drug Development, Pusan National University, Busan 46241, Korea; 3 Jeju Research Institute of Pharmaceutical Sciences, College of Pharmacy, Jeju National University, Jeju 63243, Korea; kwondoy@jejunu.ac.kr; 4 Healthcare R&D Center, HP&C Ltd., Cheongju 28158, Korea; dyyuk@hpnc.co.kr

**Keywords:** endoplasmic reticulum stress, glutathione, lipid accumulation, non-alcoholic fatty liver disease, oxidative stress

## Abstract

Non-alcoholic fatty liver disease (NAFLD) is a chronic liver dysfunction characterized by excess lipid accumulation; non-alcoholic steatohepatitis can transform into more severe conditions, such as cirrhosis and hepatocellular carcinoma. Although several pharmacologic approaches have been evaluated in clinical trials, there are no approved therapies for NAFLD. Previous studies have suggested that taurine supplementation alleviates fatty liver; however, the underlying mechanism remains obscure. In this study, we investigated the beneficial effects of taurine on fatty liver injury in vivo induced by tunicamycin, a chemical endoplasmic reticulum (ER) stressor. The mice were administered 2% taurine for 2 weeks prior to intraperitoneal tunicamycin injection; after 72 h of treatment, the mice were euthanized. Tunicamycin treatment significantly increased the levels of serum ALT and AST and hepatic triglycerides. Notably, these changes were alleviated by taurine supplementation. Taurine normalized the protein and/or mRNA levels involved in ER stress signaling (IRE1a, p-IRE1a, ATF6, XBP1, BiP, and CHOP) and lipid metabolism (CD36, MTTP, and ApoB), which were dysregulated by tunicamycin treatment. The stimulation of hepatic lipid export by taurine was evidenced by the recovery of blood VLDL levels. Furthermore, taurine supplementation prevented tunicamycin-induced lipid peroxidation and decreased glutathione (GSH) levels by correcting abnormal cysteine catabolism involved in the production of both taurine and GSH. Therefore, taurine supplementation can prevent tunicamycin-induced liver injury by counteracting oxidative and ER stress.

## 1. Introduction

Non-alcoholic fatty liver disease (NAFLD) is a form of hepatic dysfunction characterized by excess lipid accumulation in the hepatocytes. It encompasses a spectrum of related diseases, from simple steatosis to non-alcoholic steatohepatitis (NASH) [1,2]. NAFLD is a well-known etiology of chronic liver disease, which is pathologically associated with obesity; it has become a rising healthcare burden affecting almost 25% of the global population and 85–98% of obese patients [3,4]. NAFLD is a multisystem disease that affects not only the liver but also extrahepatic organs and regulatory pathways, thereby elevating the risks for type 2 diabetes, cardiovascular diseases, and chronic kidney disease. This condition can further progress to severe liver diseases, such as cirrhosis and liver cancer [5,6]. Pharmacotherapeutic approaches, including metformin, vitamin E, ursodeoxycholic acid, and delayed-release cysteamine, have been considered as optimal treatment agents. However, the efficacy of these substances has not been proven and remains to be unbeneficial; lifestyle modification remains to be the cornerstone of primary NAFLD treatment [7,8]. Thus, this observation implies the scarcity of research pertaining to the validation of predictive disease biomarkers, response to therapy, and the need for therapeutic strategies [4].

An imbalance between lipid acquisition (i.e., fatty acid uptake and de novo lipogenesis) and removal (i.e., mitochondrial fatty acid oxidation and extrahepatic lipoprotein transport) leads to triglyceride accumulation in the cytoplasm of hepatocytes, which is a hallmark of NAFLD [5]. Furthermore, a variety of risk factors in an individual, including oxidative stress, pro-inflammatory cytokines, and disruptions in lipid metabolism, are known to stimulate the development of NAFLD [9]. However, its associated mechanisms of disease progression are yet to be elucidated [1]. Among various factors, dysfunction of the endoplasmic reticulum (ER) is suggested as an important factor correlating excess fatty acids and liver damage [3].

In eukaryotic cells, the endoplasmic reticulum (ER) is the largest organelle that facilitates the synthesis, folding, and transport of proteins. High-quality protein folding in the ER ensures normal organismal physiology; conversely, disturbed ER homeostasis causes the accumulation of unfolded or misfolded proteins in the ER lumen, which is the main mechanism of ER stress. ER stress under severe or unresolved conditions triggers inflammation and promotes cell death [10,11,12,13]. It is also closely related to oxidative stress; protein misfolding results in the production of reactive oxygen species (ROS), thereby disturbing the redox state and deteriorating the protein folding process [14]. ER is a primary site for lipid metabolism and homeostasis, owing to the presence of enzymes [15,16]; thus, aberrant lipid changes in hepatocytes during hepatic steatosis can further induce chronic ER stress in the liver [1]. Therefore, it can be suggested that ER and oxidative stress, as well as abnormal lipid homeostasis in the liver, can stimulate the development of NAFLD.

Taurine (2-aminoethanesulfonic acid) is a primary intracellular, sulfur-containing substance that can be supplied via dietary ingestion or derivation from other sulfur-containing amino acids, such as cysteine and methionine [17]; these substances play various roles in certain physiologic processes, such as osmoregulation, anti-inflammation, calcium homeostasis, bile salt formation, and central nervous system function [9,18,19,20]. In particular, taurine is considered a cytoprotective molecule due to its capability of maintaining glutathione (GSH) stores, scavenging ROS, and alleviating ER stress. Furthermore, taurine-based treatments alleviate the aggravating effects of mitochondrial oxidative stress by normalizing GSH, an endogenous antioxidant that protects cells from ROS. It has been theorized that taurine supplementation causes GSH augmentation by facilitating cysteine into the GSH synthesis pathway [21,22]. In addition, taurine plays a role in repairing glutamate toxicity by suppressing ER stress-mediated apoptosis [21]. Studies have reported that taurine plays an important role in maintaining normal lipid metabolism, which may contribute to its remedial effects on NAFLD [19,23]. Furthermore, studies have shown that taurine alleviated steatosis and inflammation in a NAFLD rat model [24] and blunted hepatic cholesteryl ester accumulation in high-cholesterol diet-fed rats [20]. It was also observed that taurine-associated mitigation of hepatic steatosis may have been due to its ability in attenuating triglyceride and cholesterol accumulation [18]. However, detailed mechanisms are yet to be elucidated. In this study, the underlying mechanisms of taurine on tunicamycin-induced hepatic steatosis were investigated to determine its beneficial effects on ER and oxidative stress as well as in lipid homeostasis.

## 2. Materials and Methods

### 2.1. Animal and Treatments

Male FVB/NHsd mice (6-weeks-old) were purchased from KOATECH (Pyeongtaek, Korea). The mice were housed in a specific animal facility with temperature (22 ± 2 °C) and relative humidity (55 ± 5%)-controlled rooms and a 12-h light/dark cycle. They were also provided with free access to water and a standard irradiated chow diet (Samtako Inc., Osan, Korea). The experimental schedule of taurine and tunicamycin treatments and their doses were decided with reference to those of previous studies [9,19,23]. Taurine-dissolved tap water (2%) replaced regular tap water for 14 days following a week of acclimatization. Then, they received a single intraperitoneal injection of tunicamycin (0.5 mg/kg); samples were collected 72 h after treatment. The animal protocols were approved and established by the Institutional Animal Care and Use Committee of Pusan National University (No. PNU-2020-2732).

### 2.2. Examination of Serum Biochemical Parameters

Blood samples were collected and transferred into a BD Microtainer Blood Collection Tube (BD Life Sciences, Franklin Lakes, NJ, USA). In order to obtain the serum; the samples were centrifuged at 4000× *g* for 20 min at 4 °C and stored at −80 °C for biochemical analyses. Serum activities of alanine aminotransferase (ALT) and aspartate aminotransferase (AST) were measured using the protocol of Reitman and Franke [25]. The absorbance was measured colorimetrically at 505 nm using a MULTISKAN GO reader (Thermo Scientific, Waltham, MA, USA).

### 2.3. Examination of Hematoxylin and Eosin Staining for Liver Histology

The liver tissue was fixed in a 10% neutral-buffered formalin solution. Following standard tissue processing, the liver tissues were embedded in paraffin for hematoxylin and eosin (H&E) staining. The tissues were sectioned (3–5 μm), mounted on glass slides, and examined under a light microscope (Olympus CX41RF, Olympus Co., Tokyo, Japan).

### 2.4. Immunoblotting Analysis

The samples were then homogenized with ice-cold ProEX™ CETi protein extract solution (TransLab Biosciences, Daejeon, Korea), which contained a protease and phosphatase inhibitor cocktail. The concentration of protein in the lysates was determined using the BCA procedure (Thermo Scientific, Sunnyvale, CA, USA). The same amounts of protein were separated and subjected to sodium dodecyl sulfate-polyacrylamide gel electrophoresis (SDS-PAGE). The samples were then transferred onto nitrocellulose (NC) membranes (Bio-Rad, Hercules, CA, USA). The membranes were incubated with 0.1% Tween-20 (TBS-T), which contained 5% skimmed milk for 30 min at 25 °C. They were then washed with TBS-T buffer. The following primary antibodies were incubated overnight at 4 °C (dilution 1:2000 to 1:5000): anti-IRE1α, anti-p-IRE1α (Cell Signaling Technology, Danvers, MA, USA), anti-ATF6, anti-CHOP, anti-GCLC, anti-GAPDH (Santa Cruz Biotechnology, Santa Cruz, CA, USA), anti-CD36 (Abcam, Cambridge, MA, USA), and anti-CDO (Abcam, Cambridge, MA, USA). The blots were washed with TBS-T and incubated with the appropriate horseradish peroxidase-conjugated secondary antibodies. The resulting antigen-antibody complexes were detected using the EZ-Western Lumi Pico detection kit (DOGEN, Seoul, Korea).

### 2.5. Real-Time Reverse Transcription Polymerase Chain Reaction (qRT-PCR)

Liver tissues were used for the determination of mRNA expression by qRT-PCR. Total RNA was isolated from 10 mg of mouse liver using GeneAll RiboEx Total RNA extraction reagent (GeneAll Biotechnology, Seoul, Korea) and Direct-zol™ RNA MiniPrep (Zymo Research, Irvine, CA, USA). Extracted RNA (2.0 μL) was converted to cDNA using the iScript cDNA synthesis kit (Bio-Rad Laboratories, Inc., Hercules, CA, USA). The qRT-PCR amplification was performed using the SensiFAST™ SYBR^®^ No-ROX Kit (Bioline, London, UK) on the CFX Connect TM Real-Time System (Bio-Rad Laboratories, Inc.). The primer sequences used in this study are listed in Table 1. Each mRNA level was normalized by that of *b-actin*.

### 2.6. Examination of Triglycerides (TG) in the Liver

Total lipids were isolated from 250 mg of mouse liver in a chloroform-methanol mixture (2:1, *v*/*v*). The total lipid extract was measured enzymatically using a commercially available enzymatic TG assay kit (AM157S-K; Asan Pharmaceutical, Seoul, Korea) according to the manufacturer’s protocol. TG concentration was measured colorimetrically at 532 nm using a MULTISKAN GO reader (Thermo Scientific, Waltham, MA, USA).

### 2.7. Examination of Oil Red O Staining

In order to measure TG accumulation in the liver tissue, 5 µm-thick cross sections of the left lateral lobe were immersed in propylene glycol for 5 min and then stained with Oil Red O (Sigma Aldrich, St. Louis, MO, USA) reagent to assess lipid staining for 7 min. After rinsing with 85% propylene glycol and distilled water, the sections were counterstained with hematoxylin for 2 min before microscopic examination.

### 2.8. Examination of Free Fatty Acids (FFA) in the Liver

FFA level in the liver was determined by using a Free Fatty Acid Assay Kit (Biomax, Seoul, Korea) according to the manufacturer’s instruction.

### 2.9. Determination of Blood Very-Low-Density Lipoprotein (VLDL) Level

Serum VLDL level was determined by using EZ-VLDL assay kit (DoGenBio Co., Ltd., Seoul, Korea).

### 2.10. Examination of Hepatic Lipid Peroxidation

Hepatic lipid peroxidation was measured using the thiobarbituric acid reactive substrate (TBARS) assay. The following experiment was performed in accordance with the Volpi and Tarugi procedures [26]. The liver was homogenized in a triple volume of 1.15% KCl. The liver lysate was mixed with 6.7% trichloroacetic acid (TCA) for 15 min on ice and centrifuged at 10,000× *g* for 15 min at 4 °C. The supernatant was mixed with an equal volume of 0.67% thiobarbituric acid (TBA). Then, the mixture was incubated at 100 °C for 10 min. The complex levels of lipid peroxidation and TBA were measured colorimetrically at 532 nm using a MULTISKAN GO reader (Thermo Scientific).

### 2.11. Examination of Sulfur-Containing Metabolites

Homogenization of liver tissue with 4 volumes of buffer (150 mM NaCl, pH 7.4) resulted in liver lysate. The liver lysate was mixed with 1 M perchloric acid, which contained either 2 mM EDTA or methanol. The denatured protein was removed by centrifugation at 10,000× *g* for 10 min at 4 °C. The supernatant was used to determine cysteine or taurine and GSH. The cysteine level was determined using HPLC with a fluorescence detector (excitation at 385 nm and emission at 515 nm; FLD-3100, Thermo Scientific, Sunnyvale, CA, USA) [27,28]. They were separated with a Hector M-C18 column (3 µm × 4.6 mm × 150 mm; Chiral Technology Korea, Daejeon, Korea) and fluorescence detector using the 7-fluorobenzofurazan-4-sulfonic acid ammonium salt (SBD-F) pre-column derivatization method [29]. GSH and oxidized GSH (GSSG) was quantified using GSH/GSSG-GloTM Assay Kit (Promega, Madison, WI, USA). Taurine was derived using o-phthalaldehyde/2-mercaptoethanol and measured using an HPLC system with a fluorescence detector. They were separated using a Hector T-C18 column (3 µm × 4.6 mm × 100 mm; Chiral Technology Korea, Daejeon, Korea)

### 2.12. Examination of Reactive Oxygen Species (ROS) Generation

Hepatic ROS generation was determined using 2′,7′-dichlorodihydrofluorescein diacetate (DCFDA, Merck KGaA, Darmstadt, Germany). The liver homogenate was incubated with DCFDA (25 µM) in phosphate buffer (50 mM), and the fluorescence was detected for 60 min at wavelengths of 480 nm (excitation) and 535 nm (emission).

### 2.13. Statistical Analysis

All results were indicated as mean ± standard deviation (SD). Means of different groups were compared using one-way analysis of variance (ANOVA) followed by Newman–Keuls multiple range test as a post-hoc analysis using GraphPad Prism version 5.0 software (GraphPad Software, San Diego, CA, USA). The acceptable level of significance was established at *p* < 0.05.

## 3. Results

### 3.1. Taurine Alleviated Tunicamycin-Induced Hepatotoxicity in Mice

**Figure 1:**
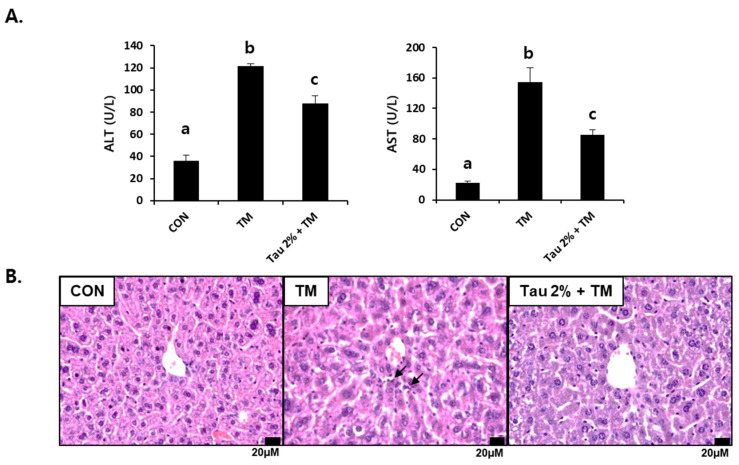
Effect of taurine on tunicamycin (TM)-induced hepatotoxicity. (**A**) Serum activities of alanine transferase (ALT) and aspartate aminotransferase (AST) in mice. (**B**) Histopathological examination of hematoxylin and eosin (H&E)-stained liver tissues at 72 h after TM treatment. Values are represented as the mean ± SD. Values with different letters (a, b, c) are significantly different from one another at *p* < 0.05 (one-way ANOVA followed by Newman-Keuls test). Arrows indicate inflammatory cells.

Both ALT and AST in tunicamycin-treated mice (0.5 mg/kg body weight) were significantly higher than those in control mice 72 h after treatment (Figure 1A). In contrast, their levels were substantially reduced in the taurine group as compared to those in the TM-treated group. We performed histological analysis following H&E staining of the liver cellular structures. The liver of tunicamycin-treated mice showed inflammatory cell infiltration, which was lowered by taurine supplementation (Figure 1B). Thus, these results suggested that taurine treatment effectively protects against tunicamycin-induced hepatic injury and inflammation.

### 3.2. Taurine Attenuated Tunicamycin-Induced ER Stress Response in the Liver

**Figure 2:**
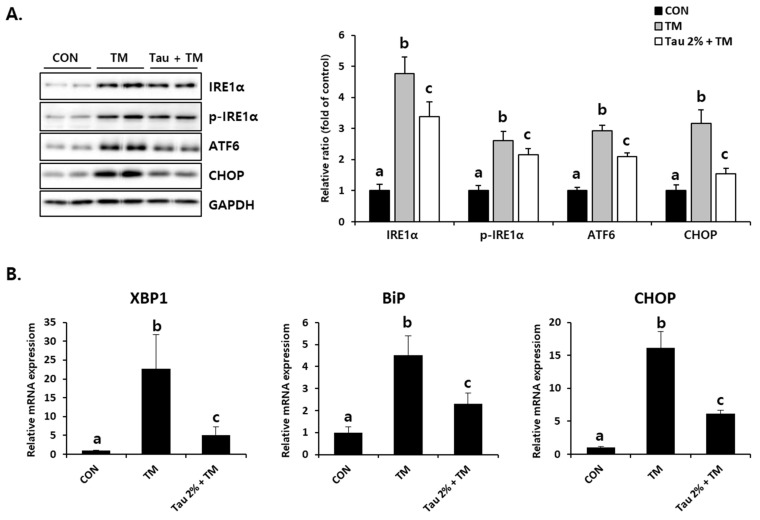
Effect of taurine on TM-induced ER stress in the livers. (**A**) Protein levels of IRE1α, phospho-IRE1α, ATF6, and CHOP (Whole blot images can be found in Figure S1). (**B**) XBP1, BiP, and CHOP mRNA expressions. Values are represented as the mean ± SD. Values with different letters (a, b, c) are significantly different from one another at *p* < 0.05 (one-way ANOVA followed by Newman-Keuls test).

To identify tunicamycin-induced ER stress responses, we initially evaluated the expression of representative ER stress markers. Disruption of homeostasis in the ER, referred to as ER stress, activates the UPR signaling system [30]. As shown in Figure 2A, the protein levels of an inositol-requiring enzyme (IRE), phospho-IRE, which is known as the UPR sensor, as well as those of activating transcription factor 6 (ATF6) and CCAAT/enhancer-binding protein homologous protein (CHOP), which are activated by ER stress, were significantly increased in the liver of tunicamycin-treated mice. Pretreatment with taurine significantly inhibited these ER stress-related proteins (Figure 2A). The mRNA expressions of X-box binding protein 1 (XBP1), binding immunoglobulin protein (BiP), and CHOP induced by tunicamycin were inhibited by taurine (Figure 2B). XBP1 promotes gene expressions involved in chaperone and lipid synthesis under ER stress, and BiP is well known ER stress marker that supports protein folding [10,15,16]. Thus, these results indicated that taurine could be a preventive supplement against ER stress.

### 3.3. Taurine Prevented Tunicamycin-Induced Lipid Accumulation in the Liver

**Figure 3:**
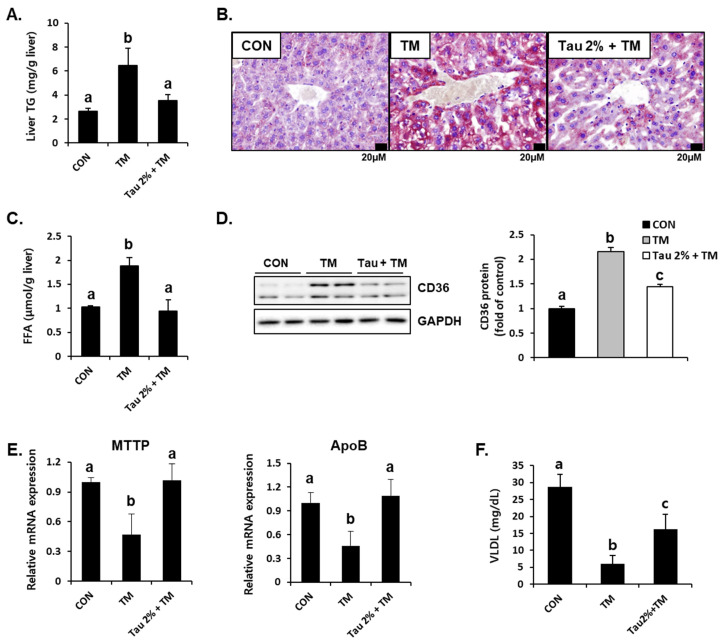
Effect of taurine on tunicamycin (TM)-induced lipid accumulation and the protein level of lipid transporter in the livers. (**A**) Levels of triglyceride (TG) in the homogenates of the liver. (**B**) Oil Red O staining in the liver. (**C**) Hepatic free fatty acid level. (**D**) Hepatic protein level of CD36 in the livers was determined by western blot analysis, and the levels of protein was normalized to that of GAPDH (Whole blot images can be found in Figure S2). (**E**) mRNA expression of microsomal triglyceride transfer protein (MTTP) and apolipoprotein B (ApoB). (**F**) Blood very-low-density lipoprotein (VLDL) level. Values are represented as the mean ± SD. Values with different letters (a, b, c) are significantly different from one another at *p* < 0.05 (one-way ANOVA followed by Newman-Keuls test).

Hepatic lipid accumulation is a common consequence of tunicamycin-induced ER stress [3,31]. To determine the effect of taurine on tunicamycin-induced lipid accumulation in the liver, we performed Oil Red O staining and determined the hepatic TG levels. Results showed that tunicamycin treatment resulted in a 3-fold increase in hepatic TG content, whereas taurine supplementation dramatically reduced TG levels (Figure 3A). Thus, Oil Red O staining confirmed a distinct difference between the taurine and tunicamycin groups (Figure 3B). Hepatic free fatty acids (FFA) levels were also elevated by tunicamycin but normalized by taurine (Figure 3C), suggesting that taurine reduced free fatty acid load in the liver.

To further support the lipid-lowering effect of taurine, we determined hepatic protein expressions involved in fatty acid uptake and lipid export [32,33]. Interestingly, the protein level of CD36, a fatty acid uptake transporter, was significantly increased in the livers of tunicamycin-treated mice (Figure 3D). Consequently, taurine treatment significantly decreased its expression (Figure 3D). Moreover, the mRNA levels of microsomal triglyceride transfer protein (MTTP) and apolipoprotein B (ApoB), which play important roles in hepatic lipid export via very-low-density lipoprotein (VLDL) synthesis, were decreased in tunicamycin-treated mice but increased in the taurine-supplemented group (Figure 3E). Tunicamycin-induced decrease of blood VLDL level was also alleviated by taurine (Figure 3F), indicating that taurine could remove hepatic lipids by VLDL-mediated export. These findings suggested that taurine protects against tunicamycin-induced hepatic lipid accumulation by regulating lipid transport.

### 3.4. Taurine Inhibited Tunicamycin-Induced Hepatic Oxidative Stress in Mice

**Figure 4:**
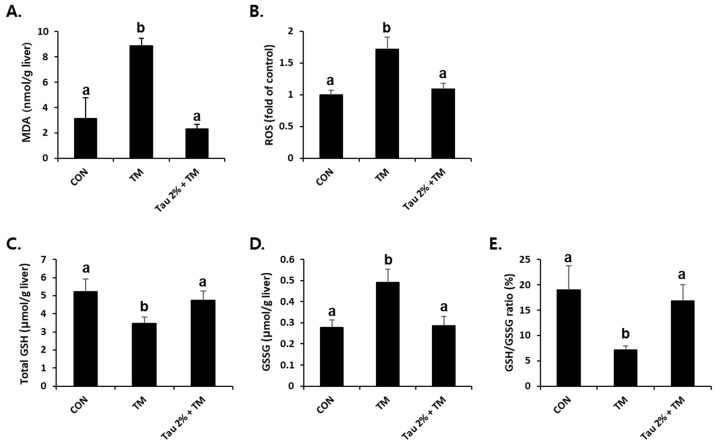
Amelioration of tunicamycin (TM)-induced oxidative stress in the livers by taurine treatment. (**A**) Malondialdehyde (MDA) levels in the livers were measured to assess lipid peroxidation. (**B**) Reactive oxygen species (ROS) generation, (**C**) total glutathione (GSH), (**D**) oxidized GSH (GSSG), and (**E**) GSH/GSSG ratio were measured in the liver tissues at 72 h after TM treatment. Values are represented as the mean ± SD. Values with different letters (a, b) are significantly different from one another at *p* < 0.05 (one-way ANOVA followed by Newman-Keuls test).

ER stress-related liver injuries are closely associated with severe hepatic oxidative stress [34]. As shown in Figure 4A,B, the tunicamycin-treated mice had higher levels of malondialdehyde (MDA), an index of lipid peroxidation, and ROS generation compared to control mice. Remarkably, taurine pretreatment effectively reduced both ROS formation and lipid peroxidation in the liver (Figure 4A,B). GSH is a key molecule responsible for endogenous antioxidant homeostasis, and the ratio of its reduced form and oxidized form (GSSG) is used as an indicator of oxidative stress. Tunicamycin-treated mice induced a hepatic redox imbalance with decreased GSH and increased GSSG contents, which were recovered after treatment with taurine (Figure 4C–E).

### 3.5. Taurine Recovered Tunicamycin-Induced Aberrant Cysteine Catabolism in the Liver

**Figure 5:**
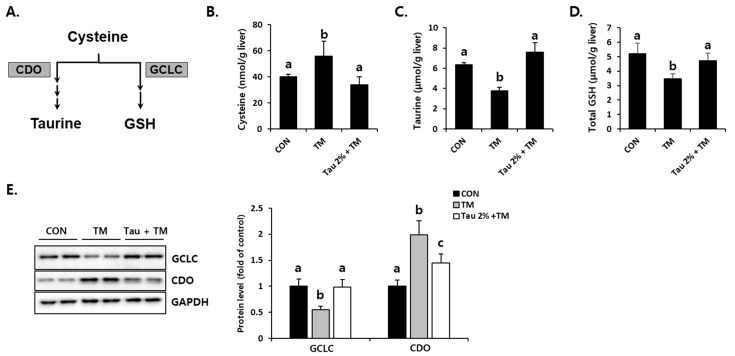
Effect of taurine pre-treatment on tunicamycin-induced abnormal cysteine catabolism in the liver. (**A**) Cysteine catabolism in the liver. (**B**) Hepatic cysteine. (**C**) Hepatic taurine (Tau). (**D**) Hepatic total GSH. (**E**) Protein levels of rate-limiting enzymes for GSH and taurine synthesis, GCLC and CDO, respectively (Whole blot images can be found in Figure S3). Hepatic substances and proteins were determined at 72 h after TM treatment. The levels of proteins were normalized by that of GAPDH. Values are represented as the mean ± SD. Values with different letters (a, b, c) are significantly different from one another at *p* < 0.05 (one-way ANOVA followed by Newman-Keuls test).

To investigate how taurine defends tunicamycin-induced oxidative stress, we focused on cysteine catabolism, which generates GSH and taurine (Figure 5A). Hepatic cysteine (precursor of GSH), taurine, and GSH levels were comparatively evaluated between taurine and tunicamycin-treated mice (Figure 5B–D). Interestingly, taurine was significantly lower in the tunicamycin treatment group than in the control group. In contrast, the taurine treatment improved tunicamycin-induced reduction in the liver. Importantly, the protein expression of CDO (Figure 5E), a rate-limiting step of taurine synthesis, was dramatically increased by tunicamycin but decreased in taurine-treated mice. Furthermore, hepatic GCLC, a rate-limiting enzyme for GSH synthesis, in the taurine-treated mice were higher than those in the tunicamycin-treated mice (Figure 5E). These data indicated that taurine protects the tunicamycin-treated mice against hepatic oxidative stress, presumably by regulating sulfur-containing substances.

## 4. Discussion

NAFLD is predicted to become the leading indication for liver transplantation by 2030 [6]. However, lifestyle modification has remained the primary option for NAFLD treatment due to a shortage of therapeutic agents [7]. Therefore, further studies are required for elucidation of underlying mechanisms and testing of new therapeutics [8]. Ingestion of taurine, an essential sulfur-containing amino acid present in mammalian tissues, alleviates metabolic diseases, such as hyperlipidemia, diabetes, hypertension, and obesity [35,36]. Although details of the mechanisms by which taurine imposes its beneficial contribution are undetermined, increasing evidence suggests that taurine administration suppresses NAFLD based on lipid profile optimization, mitigation of hepatic oxidative stress, and hepatic GSH enhancement [37]. In addition, several studies have shown that taurine suppresses the onset of liver steatosis in a rat model of NASH accompanied by lipid and glucose metabolic amelioration and alleviates inflammation and fibrosis [38]. In the present study, the therapeutic potential of taurine was investigated specifically to identify a more effective approach for NAFLD.

Tunicamycin, a well-known pharmacological ER stressor, has been used to create a NAFLD model in mice [39,40,41]. In this study, we found that taurine alleviates tunicamycin-induced ER and oxidative stress, thereby preventing the development of NAFLD. Importantly, the novelty of the current study lies in the fact that taurine disrupted the chain reactions of both ER and oxidative stress, eventually leading to hepatic recovery. This may provide new insights into the mechanism of action of taurine as a potential therapeutic agent in NAFLD.

The unfolded protein response (UPR) is a protein homeostasis-maintaining system that monitors ER conditions by sensing the inadequacy in the protein folding capacity of the ER [15,16]. Not only is the ER a protein homeostasis-maintaining system, but it is also the primary site of lipid metabolism since numerous enzymes associated with lipid metabolism are found within the organelle [15]; thus, the UPR plays vital roles in maintaining metabolic and lipid homeostasis [16]. In the liver, lipid accumulation caused by ER stress mainly occurs through mechanisms such as promoted lipogenesis, augmented lipolysis, increased fatty acid/lipoprotein uptake, reduced VLDL secretion, and decreased fatty acid oxidation, with various contributing factors for ER stress stimuli [31,42]. Our results showed that taurine supplementation significantly prevented the induction of ER stress signaling (IRE1a, ATF6, XBP1, BiP, CHOP) as well as lipid uptake transporter expression (CD36) induced by tunicamycin treatment, which contributes to the inhibition of lipid accumulation in the liver. Specifically, CD36 promotes the import of fatty acids; its expression is regulated by the liver X receptor, pregnane X receptor, and peroxisome proliferator-activated receptor (PPAR) γ [43]. Other studies using animal models of NAFLD suggested a causal role of CD36 in hepatic lipid accumulation, which were supported by a positive correlation between the expression level of CD36 and the extent of hepatic lipid accumulation [44,45]. Thus, the downregulation of CD36 by taurine in our results implicated its possibility of being an important modulator of lipid metabolism. The maintenance of ER homeostasis is important for hepatic lipid export because ER is the site of lipoprotein assembly facilitated by MTTP for VLDL synthesis [3]. Thus, the recovery of MTTP and ApoB expressions by taurine might be associated with the reduced ER stress, and the stimulation of lipid export via VLDL secretion appears to be another mechanism of the anti-steatogenic effect of taurine.

In order for the folding of proteins to go through the correct conformations as well as for the formation of intramolecular and intermolecular disulfide bonds in the ER, redox-balanced environments and calcium levels in its lumen are important. [46]. However, misfolded proteins in the ER can cause calcium leakage, thereby disrupting the electron transport chain and ROS production. Additionally, ROS production in the mitochondria can cause protein misfolding [47]. This indicates that protein folding and ROS generation as a byproduct of oxidative protein folding in the ER are closely related to one another [34,48]. Oxidative stress, defined as the perturbed balance between the generation of ROS (free radicals) and antioxidant defenses, is considered as one of the central mechanisms that induce hepatic injury in NAFLD, thereby leading to a pathological progression from simple steatosis to NASH [49,50].

In our study, tunicamycin treatment increased lipid peroxidation and reduced GSH levels, reflecting oxidative stress in the liver. However, taurine administration significantly mitigated tunicamycin-mediated oxidative stress. Notably, modulation of tunicamycin-induced aberrant cysteine catabolism could contribute to the replenishment of GSH, leading to defense against oxidative stress. The liver plays a fundamental role in the metabolism of sulfur-containing amino acids; approximately 50% of daily methionine intake is metabolized in the liver [51]. GSH, a final byproduct of sulfur-containing amino acid metabolism, is not only a powerful antioxidant but also a principal redox buffer in the ER; thus, depletion of reduced glutathione can cause additional oxidative stress [48,52]. Cysteine, the metabolic precursor of GSH, is also an essential substrate for taurine synthesis. Therefore, the utilization of cysteine for the generation of GSH and taurine is competitive [27,53,54]. In this study, taurine supplementation restored GSH levels, which were attenuated by tunicamycin treatment. This result can be accounted for by an increase in the expression of GCLC, an enzyme mediating GSH synthesis; the availability of cysteine is favored for GSH synthesis due to sufficient taurine supply.

Therefore, we speculate that the protective effect of taurine on tunicamycin-induced hepatic injury results from its concurrent mitigation of both ER and oxidative stress, disrupting the vicious cycle of pathogenesis. Downregulation of the lipid uptake transporter along with ER stress and stimulation of lipid export by VLDL secretion may contribute to decreased hepatic lipid accumulation in tunicamycin-treated mice. Our results elucidate the mechanisms by which taurine exerts its beneficial effects on liver disease, highlighting its therapeutic potential for enabling recovery from NAFLD.

## Figures and Tables

**Table 1:** List of murine primers used for real time RT-PCR.

Symbol	Primer Sequence (5′-3′)	
	Forward	Reverse
MTTP	CTCTTGGCAGTGCTTTTTCTCT	GAGCTTGTATAGCCGCTCATT
ApoB	TTGGCAAACTGCATAGCATCC	TCAAATTGGGACTCTCCTTTAGC
XBP1	GAGTCCGCAGCAGGTG	GTGTCAGAGTCCATGGGA
BiP	ATCAGGGCAACCGCATCAC	TGATGTCCTGCTGCACCGAA
CHOP	CACGCACATCCCAAAGCC	GGGCACTGACCACTCTGTT
β-actin	CTGTCCCTGTATGCCTCTG	ATGTCACGCACGATTTCC

## Data Availability

The data presented in this study are available in this article.

## Supplementary Materials

The following are available online at https://www.mdpi.com/article/10.3390/life12030354/s1, Figure S1: Whole blot images of Figure 2A, Figure S2: Whole blot images of Figure 3D. Figure S3: Whole blot images of Figure 5E.
