# Supplementary material for: Taurine Ameliorates Tunicamycin-Induced Liver Injury by Disrupting the Vicious Cycle between Oxidative Stress and Endoplasmic Reticulum Stress
Source: Life (Basel). 2022 Feb 28;12(3):354. doi: 10.3390/life12030354 (PMC8951380; doi:10.3390/life12030354)
Supplement: Supplementary file 1 [file life-12-00354-s001.zip › life-1596022-supplementary.pdf]

Supplementary Materials of Taurine Ameliorates Tunicamycin-Induced Liver Injury by Disrupting the Vicious Cycle between Oxidative Stress and Endoplasmic Reticulum Stress

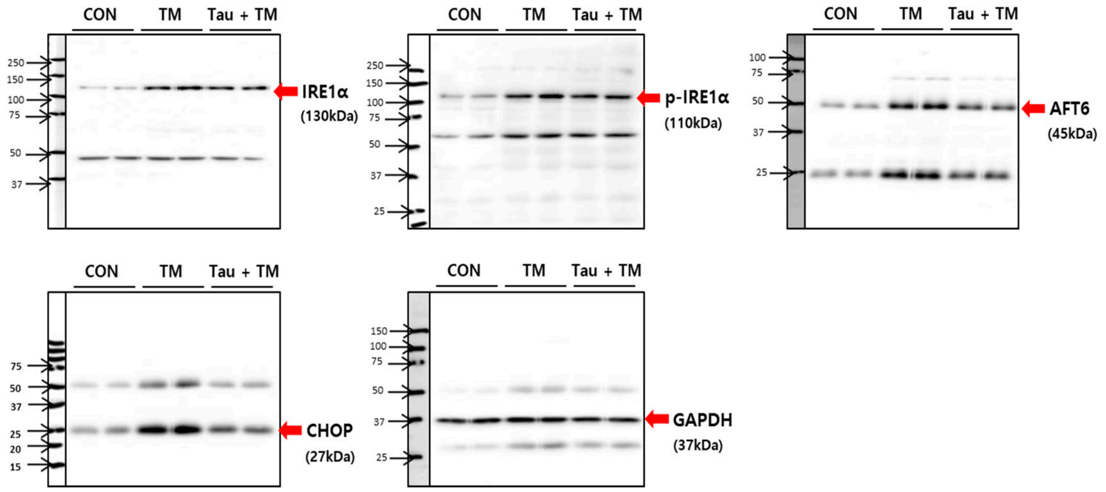

Figure S1. Whole blot images of Figure 2A.

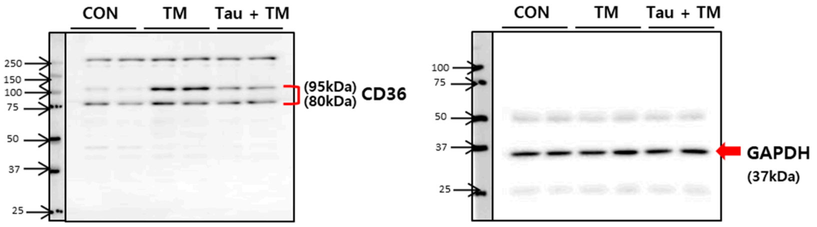

Figure S2. Whole blot images of Figure 3D.

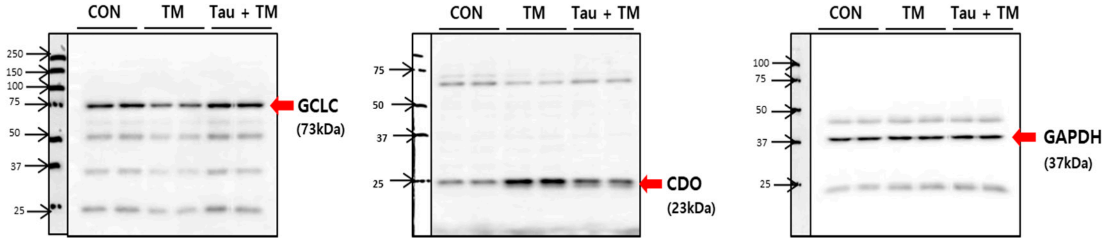

Figure S3. Whole blot images of Figure 5E.
